# Draft genome sequence of glyphosate-degrading *Ochrobactrum* sp. strain GPK 3 (VKM B-2554D) isolated from agricultural soil

**DOI:** 10.1128/mra.01186-24

**Published:** 2025-01-15

**Authors:** Alexey V. Sviridov, Sergey V. Tarlachkov, Dmitry O. Epiktetov, Tatyana V. Shushkova, Marina P. Petrakova, Alexey A. Leontievsky

**Affiliations:** 1 G.K. Skryabin Institute of Biochemistry and Physiology of Microorganisms, Pushchino Scientific Center for Biological Research of the Russian Academy of Sciences, Federal Research Center, Pushchino, Russia; 2 Branch of Shemyakin and Ovchinnikov Institute of Bioorganic Chemistry of the Russian Academy of Sciences, Pushchino, Russia; Queens College Department of Biology, Queens, New York, USA

**Keywords:** *Ochrobactrum*, glyphosate, draft genome, C-P lyase, phosphonatase

## Abstract

The genome of *Ochrobactrum* sp. isolated from agricultural soil polluted with the herbicide glyphosate is reported. The genome size is 5.48 Mb with an average G + C content of 56.3%. This genomic data could contribute to a better understanding of biochemistry and regulatory mechanisms of microbial degradation of glyphosate and 2-aminoethylphosphonic acid

## ANNOUNCEMENT

*Ochrobactrum* sp. strain GPK 3 is a bacterium isolated from soils subject to long-term glyphosate (GP) treatments in the Krasnodar region, Russia, at 45°03′10.8″N 38°52′22.8″E. Samples were incubated for 3 months at 28°C with periodic stirring and moistening with medium MS1 (1) containing GP (18 mg kg^−1^ soil), inoculated into liquid MS1 medium with 10 g L^−1^ of glutamate and 0.5 g L^−1^ of GP, and incubated on a shaker with passages twice a week, 20 in total. Resulting associations were inoculated on LB agar plates; cultures were isolated from single colonies according to morphological distinctiveness.

*Ochrobactrum* sp. GPK 3 is Gram-negative motile short rods approx. 1.1–1.6 × 0.7 µm, with temperature optimum of 28°C–30°C, forming glistening, mucoid, cream-colored colonies. The strain utilized several organophosphonates (OPs) as phosphorus sources (2) and was maintained on MS1 agar plates supplemented with 10 g L^−1^ of glutamate and 0.5 g L^−1^ of GP.

A single colony of *O. anthropi* GPK 3 from the MS1 agar plate was inoculated into liquid LB medium and cultivated on a shaker overnight at 28°C. FastDNA SPIN Kit and FastPrep-24 homogenizer (MP Biomedicals, USA) were used for DNA isolation. The short-read library was prepared with KAPA HyperPlus Kit (Roche, Switzerland) in the following steps: enzymatic DNA fragmentation, end repair and A-tailing, ligation of specific adapters, amplification of the library (seven cycles), and then sequenced on an Illumina NovaSeq 6000 platform (Illumina, USA) with NovaSeq 6000 S2 Reagent Kit to obtain 2 × 100 bp reads.

Default settings were used for software unless stated otherwise. The quality of the reads was checked with FastQC 0.12.1 (3). Adapter sequences and low-quality regions were cut with Trimmomatic 0.39 (4) with the following options: ILLUMINACLIP:TruSeq3-PE-2.fa:2:30:10; SLIDINGWINDOW:4:15; and MINLEN:30. Clean reads were assembled using SPAdes 3.15.5 (5) with options: --cov-cutoff, auto; and --careful. The quality of assembly was assessed with QUAST 5.2.0 (6). Genome annotation was performed with the NCBI PGAP 6.7 (7). Phylogeny of the isolate was confirmed by TYGS (8).

A total of 5.21 × 10^6^ paired-end reads with an average of 99.2 bp long (517 Mb) were obtained. Clean 5.17 × 10^6^ reads with an average of 98.7 bp long (510 Mb) were assembled into 171 scaffolds with 92-fold coverage. The scaffold N50 is 218,196 bp, and the largest scaffold is 629,297 bp. The genome size is 5.48 Mb with an average G + C content of 56.3%. A total of 5,061 protein-coding genes (606 coding hypothetical proteins), 51 tRNAs, 3 rRNAs, and 4 ncRNAs were predicted.

The presence of C–P lyase and phosphonatase in *Ochrobactrum* sp. GPK 3 was reported earlier (2). Operons comprising genes *phnJ* (9) and *phnX* were discovered; the latter included the gene of a novel flavine oxidase (10). This makes *Ochrobactrum* sp. GPK 3 is a promising candidate for studies of OP metabolism and the development of bioremediation techniques for OP-polluted environments.

## Data Availability

The raw reads were deposited to the NCBI SRA under accession no. SRR30504127; the draft genome project was deposited in DDBJ/ENA/GenBank under accession no. JBBYKX000000000.1. The version described in this paper is the first version, JBBYKX010000000.
